# Impact of Incentives and Contact Attempts on Recruitment of Long-Term Care Workforce

**DOI:** 10.1093/geroni/igaf122.2425

**Published:** 2025-12-31

**Authors:** Soojeong Han, Gregory Alexander, Lusine Poghosyan, M Schrimpf, Sabrina Tasnova, Racheal Rodriguez, Hana Amer

**Affiliations:** Columbia University, New York, New York, United States; Columbia University, New York, New York, United States; Columbia University, New York, New York, United States; Columbia University, New York, New York, United States; Columbia University, New York, New York, United States; Columbia University, New York, New York, United States; Columbia University School of Nursing, New York, New York, United States

## Abstract

Depending on research topic, population, and setting, the ideal and reasonable amount of incentives and number of contact attempts are different, and even more than ten times of attempts is needed. The purpose of the parent study is to examine the impact of health information technology from the perspectives of the long-term care workforce. We encountered recruitment challenges and decided to contact participants multiple times and increase incentives to improve survey responses. This report aims to assess the trend of the survey response process among nursing home (NH) administrators. Out of 392 health information technology (HIT) maturity surveys of NH administrators who responded ‘yes’ to complete the survey, 206 HIT maturity surveys were returned, and 186 were not returned. Of the 206 administrators who returned the survey, 41 returned without claiming any incentive, 143 received $25, 5 received $50, and 17 received $75. Our study demonstrates that approximately 64% (n = 132) returned the survey before attempts. After up to 3 attempts, 88.83% (n = 183) returned the survey. Beyond four attempts, survey return rate did not increase regardless of increased incentive. A Chi-square test revealed a significant association between the category of attempt number (Low: 0-3 vs. Medium/High:4-10+) and survey return, χ²(1, N = 392) = 20.09, p < .0001. Future research can build upon the findings of this study by considering contact attempts and incentives to balance recruitment efforts with the pressure or burden of multiple contact attempts during recruitment and not to compromise the voluntary nature of participation.

